# Decreased expression of microRNA-21 correlates with the imbalance of Th17 and Treg cells in patients with rheumatoid arthritis

**DOI:** 10.1111/jcmm.12353

**Published:** 2014-08-28

**Authors:** Liyang Dong, Xuefeng Wang, Jun Tan, Hao Li, Wei Qian, Jianguo Chen, Qiaoyun Chen, Jun Wang, Wenlin Xu, Caihua Tao, Shengjun Wang

**Affiliations:** a Department of Central Laboratory, The Affiliated People’s Hospital, Jiangsu UniversityZhenjiang, Jiangsu, China; b Department of Rheumatology, The Affiliated People’s Hospital, Jiangsu UniversityZhenjiang, Jiangsu, China; c Department of Central Laboratory, The Affiliated Fourth Hospital of Jiangsu UniversityZhenjiang, China

**Keywords:** rheumatoid arthritis, Treg, Th17, miRNA, pro-inflammatory cytokine

## Abstract

The imbalance of Th17/Treg cell populations has been suggested to be involved in the regulation of rheumatoid arthritis (RA) pathogenesis; however, the mechanism behind this phenomenon remains unclear. Recent studies have shown how microRNAs (miRNAs) are important regulators of immune responses and are involved in the development of a variety of inflammatory diseases, including RA. In this study, we demonstrated that the frequencies of CD3^+^CD4^+^IL-17^+^Th17 cells were significantly higher, and CD4^+^CD25^+^FOXP3^+^ Treg cells significantly lower in peripheral blood mononuclear cells from RA patients. Detection of cytokines from RA patients revealed an elevated panel of pro-inflammatory cytokines, including IL-17, IL-6, IL-1β, TNF-α and IL-22, which carry the inflammatory signature of RA and are crucial in the differentiation and maintenance of pathogenic Th17 cells and dysfunction of Treg cells. However, the level of miR-21 was significantly lower in RA patients, accompanied by the increase in STAT3 expression and activation, and decrease in STAT5/pSTAT5 protein and *Foxp3* mRNA levels. Furthermore, lipopolysaccharide stimulation up-regulated miR-21 expression from healthy controls, but down-regulated miR-21 expression from RA patients. Therefore, we speculate that miR-21 may be part of a negative feedback loop in the normal setting. However, miR-21 levels decrease significantly in RA patients, suggesting that this feedback loop is dysregulated and may contribute to the imbalance of Th17 and Treg cells. MiR-21 may thus serve as a novel regulator in T-cell differentiation and homoeostasis, and provides a new therapeutic target for the treatment of RA.

## Introduction

Rheumatoid arthritis (RA) is a systemic, inflammatory, autoimmune disorder with progressive articular damage that may result in lifelong disability. Although major strides in understanding the disease have been made, the intricate mechanistic details behind the pathogenesis of RA have not yet been fully elucidated 1. It is largely accepted that an important role is played by the immune system, where synovia are infiltrated with macrophages and CD4^+^ T lymphocytes 2. CD4^+^ Helper T cells clearly play a central role in the initiation and perpetuation of chronic inflammation prevalent in RA. According to the cytokine microenvironment in which they are situated, CD4^+^ T cells differentiate towards various pro- and anti-inflammatory subpopulations, including Th1, Th2, Th17 and T regulatory (Treg) cells. It has been shown that active RA results from an imbalance in the distribution of pro-inflammatory Th17 and anti-inflammatory Treg cells, which emphasizes the crucial roles of these cells in controlling RA 3. Elevated levels of Th17 cells as well as defects in Treg cells have been observed in RA patients 4,5. However, the mechanisms governing the imbalance of Th17/Treg cells resulting in RA remains unclear.

Recently, several studies highlighted that microRNAs (miRNAs) are important regulators of the immune response 6. Several miRNAs, including miR-155 7, miR-146a 8 and miR-326 9, affect the functions of T and B cells, thereby modulating autoimmune pathogenesis 10. Furthermore, miRNAs are involved in crucial cellular processes and their dysregulation has been described in RA. Stanczyk *et al*. revealed a prominent up-regulation of miR-155 and miR-146a in RA synovial fibroblasts compared with those in patients with osteoarthritis 11. More recently, a significant increase in miR-146a, miR-155, miR-132 and miR-16 in peripheral blood mononuclear cells from RA patients compared with healthy and diseased control individuals has been highlighted, suggesting that miRNAs can be involved at different levels in the regulation of RA pathogenesis 12.

MicroRNA-21 (miR-21) is the most commonly up-regulated microRNA in a variety of cancers 13. Recently, many studies reported that miR-21 is involved in the development of inflammatory diseases and T-cell differentiation 14–16. miR-21 is increased in allergic diseases in both mouse and human 17, promotes Th2 responses by inhibiting IL-12 in myeloid cells 18, and is expressed at higher levels in Tregs compared to conventional CD4^+^CD25^−^ T cells 19; but the functional significance of Treg-specific expression of miR-21 has not been ascertained. Interestingly, STAT3, a transcription factor involved in tumorigenesis 20, which also acts as a master regulator that directs the differentiation program of Th17 cells 21, is a miR-21 target 22,23. STAT3 can also regulate miR-21 expression 24. Furthermore, STAT5, as a key transcription factor in Treg cell differentiation, can bind to the promoter region of miR-21 and up-regulate miR-21 expression, which is involved in the regulation of the balance between tolerance and immune activation 25. Thus, we has been suggested that miR-21 may be involved in the imbalance of Th17/Treg cells in RA, which has not yet been reported in the literature.

In this study, we found that the increase in Th17 cells and decrease in Treg cells in peripheral blood mononuclear cells (PBMC) from RA patients was accompanied by an increase in pro-inflammatory cytokines, including IL-17, IL-6, IL-1β and TNF-α. In addition, miR-21 was down-regulated, alongside the increase in STAT3, and decrease in STAT5/pSTAT5 in RA patients. We thus propose that miR-21 may be involved in the imbalance of Th17/Treg cells through regulating STAT3/STAT5 in RA patients.

## Materials and methods

### Patients

Twenty-five patients with active RA, fulfilling the criteria of the American college of Rheumatology 26, and twenty age- and sex-matched healthy controls were enrolled into this study. Disease activity was assessed using the 28-joint disease activity score (DAS28) 27 and all patients were free of infectious diseases, malignant diseases, cardiovascular complaints or other inflammatory diseases. The characteristics of RA patients and healthy controls were summarized in Table 1. Informed consent was obtained from all patient and healthy controls, and the study was approved by the ethics committee of the Affiliated People’s Hospital, Jiangsu University (Permit Number: JSU 11-0012), which abides by the Helsinki Declaration on ethical principles for medical research involving human participants.

**Table 1:** Characteristics of RA patients and healthy controls

Demographics	Active RA (*n* = 25)	Healthy (*n* = 20)
Age (years), mean (range)	51.2 (32–70)	50.8 (35–71)
Sex, female/male	21/4	17/3
Disease duration (years), mean (range)	8.4 (1–22)	–
RF, positive/negative	16/9	–
ESR (mm/h), mean (range)	49.4 (27–79)	8.4 (4–19)
CRP (mg/L), mean (range)	60.3 (22–149)	2.8 (1–7)
Mean DAS28	5.6 (4.4–7.2)	–
Treatment, +/−	13/12	–

*RF* Rheumatoid factor, *ESR* Erythrocyte sedimentation rate, *CRP* C-reactive protein; *DAS28* Disease Activity Score in 28 joints.

### Cell extraction and stimulation

Peripheral blood mononuclear cells were isolated from healthy controls and RA patients by Ficoll-Hypaque (Amersham Pharmacia Biotech, Uppsala, Sweden) density gradient centrifugation. The cells were washed three times with sterile PBS and suspended in a concentration of 2 × 10^6^ cells/ml in RPMI 1640 (Life Technologies, Grand Island, NY, USA) supplemented with 10% heat-inactivated foetal calf serum, 2 mM L-glutamine, and 1% penicillin-streptomycin (HyClone, Logan, UT, USA). CD4^+^ T cells were sorted from PBMC using EasySep™ Human CD4^+^ T-cell negative-isolation kit (StemCell Technologies, Vancouver, BC, Canada) according to the manufacturer’s instruction. The purity of CD4^+^ T cells was >95% by Flow Cytometry analysis.

Peripheral blood mononuclear cells were stimulated in a concentration of 2 × 10^5^ cells per well in 200 μl complete culture medium with 5 μg/ml phytohaemagglutinin (PHA) or 1 μg/ml lipopolysaccharide (LPS, *Escherichia coli* O127:B8E, L4517; Sigma-Aldrich, St Louis, MO, USA) in U bottom 96-well plates at 37°C. After 24 hrs, the cell culture supernatants were collected to quantitatively measure IFN-γ, TNF-α, IL-17, IL-6, IL-1β, IL-22 and IL-10 production using the FlowCytomix Human Cytokine Kit (Bender MedSystems, Vienna, Austria) according to the manufacturer’s instructions, and the cell pellets of the stimulated PBMC were collected to detect mRNA expression of *Il-23* and *Il-12p35* by real-time PCR as described.

To assess the miR-21 expression or phosphorylated proteins after LPS stimulation, PBMC from RA patients and healthy controls were cultured in the presence of 200 ng/ml LPS at 1 × 10^6^ cells per well in 24-well plates for 24 hrs at 37°C. The cells were collected and washed with sterile PBS to extract RNA for detecting miR-21 by real-time PCR, or to extract protein for detection by Western blotting.

### Flow Cytometry

For analysis of Th17 cells, PBMC were suspended at a density of 1 × 10^6^ cells/ml in complete culture medium (RPMI-1640 supplemented with 10% heat-inactivated foetal calf serum) for 5 hrs, in the presence of phorbol myristate acetate (25 ng/ml) plus ionomycin (1 μg/ml) and brefeldin A (1 μg/ml), at 37°C in 5% CO_2_. The cells were then incubated with human APC-anti-CD3 and FITC-anti-CD8a mAbs, washed, fixed, and permeabilized with Cytofix/Cytoperm (BD PharMingen, San Diego, CA, USA). Cells were then intracellularly stained with PE-anti-IL-17A or PE-conjugated rat IgG1 (isotype control) for 1 hr at room temperature.

For analysis of Treg cells, PBMC without stimulation were surface-stained with human FITC-anti-CD4 mAb, and APC-anti-CD25 mAb, followed by fixation and permeabilization with Cytofix/Cytoperm and intracellular staining with PE-anti-Foxp3 or PE-IgG2a rat IgG control antibody according to the manufacturer’s instructions. Data were collected on a FACSCalibur flow cytometer using CellQuest software (BD Biosciences, Franklin Lakes, NJ, USA).

### RNA isolation and quantitative real-time PCR (qRT-PCR)

Total RNA was extracted from individual PBMC, or CD4^+^ T cells using Trizol reagent (Invitrogen, Carlsbad, CA, USA), according to the manufacturer’s protocols. For assessment of miR-21, 1 μg of total RNA from PBMC of RA patients and healthy controls, CD4^+^ T cells, or PBMC after LPS-stimulated was reverse transcribed using the All-in-one™ miRNA qRT-PCR Detection Kit (Genecopoeia, Germantown, MD, USA) according to the manufacturer’s instructions. Expression of miR-21 was detected by qRT-PCR using All-in-one™ miRNA qPCR Primers (cat. no. HmiRQP0316). The relative levels of miR-21 transcripts were normalized to control U6 (cat. no. HmiRQP9001). For measurement of *Il-23*, *Il-12p35*, *Pdcd4*, *Stat3*, *Stat5a*, *Stat5b*, *Rorc* and *Foxp3* mRNA, 500 ng of total RNA from PBMC of RA patients and healthy controls, stimulated or not with LPS, were reverse transcribed using the All-in-one™ First-Strand cDNA Synthesis kit (Genecopoeia) according to the manufacturer’s manual. Real-time PCR was performed with All-in-one™ qPCR Mix (Genecopoeia) in a CFX96™ Real-Time system (Bio-Rad Laboratories Inc, Hercules, CA, USA). All-in-one™ qPCR Primer sets for *Il-23* (cat. no. HQP012859), *Il-12p35* (cat. no. HQP009692), *Pdcd4* (cat. no. HQP007623), *Stat3* (cat. no. HQP017767), *Stat5a* (cat. no. HQP017771), *Stat5b* (cat. no. HQP017774), *Rorc* (cat. no. HQP016378) and *Foxp3* (cat. no HQP012269) (Genecopoeia) were used, and human GAPDH (cat. no. HQP006940) was used as an endogenous control for sample normalization. Thermocycler conditions comprised an initial holding at 95°C for 10 min., which was followed by a 3-step PCR programme at 95°C for 10 sec., 60°C for 20 sec. and 72°C for 15 sec. for 40 cycles. Data analyses were conducted by the comparative Ct method as described previously 9,28.

### Western blotting

Peripheral blood mononuclear cells from RA patients and healthy controls, with or without stimulation by LPS, were collected and washed three times with sterile PBS. For total cellular protein, cells were lysed with RIPA Lysis Buffer (Beyotime, Nantong, China), supplemented with PMSF (Beyotime) and Protein phosphatase inhibitors (Boster, Wuhan, China). The proteins were quantitated using Thermo NanoDrop 1000 (Thermo Fisher Scientific, Wilmington, DE, USA). Equal amounts of proteins (200 μg) were electrophoresed in 10% sodium dodecyl SDS-PAGE and transferred onto polyvinylidene difluoride membranes (Bio-Rad) pre-soaked with 100% methanol. Non-specific binding was blocked for 1 hr at room temperature in Tris-buffered saline/0.05% Tween-20 (TBST) containing 5% non-fat milk powder, followed by incubation with the rabbit anti-human Ab against STAT3, STAT5, phospho-STAT3 (Tyr705, p-STAT3), phospho-STAT5 (Tyr694, p-STAT5) and GAPDH (Cell Signaling Technology Inc, Beverly, MA, USA) overnight at 4°C. Blots were washed with TBST, and incubated with horseradish peroxidase-conjugated anti-rabbit IgG secondary Ab (Cell Signaling Technology) for 1 hr at room temperature. Finally, immunoblot signals were visualized using BeyoECL Plus (Beyotime), then imaged and quantitated using a ChemiScope 3400 Mini (CLINX Science Instruments, Shanghai, China).

### Statistical analysis

Statistical analyses were performed with GraphPad Prism 5.01 (GraphPad Software, 2007, La Jolla, CA, USA). Data are expressed as the mean ± SEM or median and IQR. The Student’s *t*-test and non-parametric Mann–Whitney *U*-test were used to calculate the significance difference between the groups and a *P* < 0.05 (two-tailed) was considered as statistically significant. The correlation between miR-21 expression and transcription factors and the percentage ratio of Th17/Treg cells in PBMCs from RA patients was analyzed using Spearman correlation and *P* < 0.05 was considered statistically significant.

## Results

### Increased Th17 and decreased Treg frequencies in PBMC of RA patients

**Figure 1:**
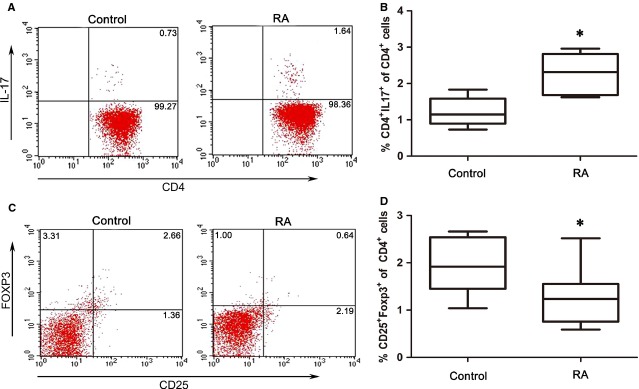
Increased circulating CD3^+^CD4^+^IL-17^+^ Th17 cells and decreased CD4^+^CD25^+^Foxp3^+^ Treg cells in rheumatoid arthritis (RA) patients. (**A**) Peripheral blood mononuclear cells (PBMC) from RA patients (*n* = 25) and healthy controls (*n* = 20) were stimulated with PMA, ionomycin and BFA for 5 hrs, and then stained with fluorescently labelled anti-human antibodies. FCM analysis for CD3, CD4 and IL-17 was performed. The representative flow cytometric results are shown, and values indicate the percentage of events in the indicated quadrant. (**B**) Collective analysis of Th17 cells from RA patients and healthy controls; data are expressed as box plots. Each box represents the interquartile range (IQR). Lines inside the boxed represent the median. Whiskers represent the highest and lowest values. *P < 0.05, *versus* control (Mann–Whitney *U*-test). (**C**) PBMC from RA patients and healthy controls were stained with labelled anti-human antibodies. FCM analysis for CD4, CD25 and Foxp3 was performed. The representative flow cytometric results are shown, and values indicate the percentage of events in the indicated quadrant. (**D**) Collective analysis of Treg cells from RA patients and healthy controls, and the data are expressed as box plots. Each box represents the IQR. Lines inside the boxed represent the median. Whiskers represent the highest and lowest values. *P < 0.05, *versus* control (Mann–Whitney *U*-test).

The frequencies of Th17 and Treg cells in PBMC from RA patients and healthy controls were determined by flow cytometry. Because PMA-ionomycin stimulation induces a decrease in surface CD4 expression, we applied CD3^+^ and CD8^−^ gates to represent the former CD4^+^ T cells 29. Consistent with results described previously 5, the proportion of CD3^+^CD4^+^IL-17^+^ Th17 cells were significantly higher in RA patients compared with the healthy controls (Fig. 1A and B). However, the proportion of CD4^+^CD25^+^FOXP3^+^ Treg cells was remarkably lower in patients with RA than the healthy controls (Fig. 1C and D). These results once again suggest that the imbalance of Th17/Treg cells exists in RA patients and is involved in RA pathogenesis.

### Elevated level of pro-inflammatory cytokines in RA patients

**Figure 2:**
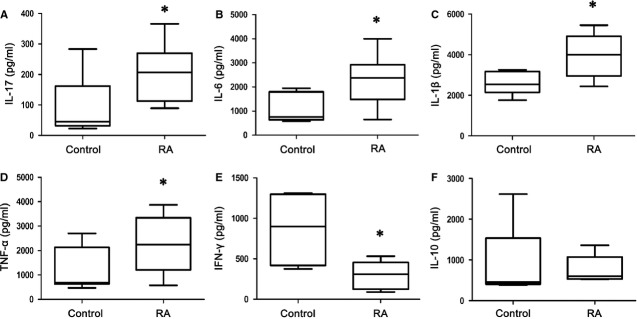
Increase in the production of pro-inflammatory cytokines from peripheral blood mononuclear cells (PBMC) with rheumatoid arthritis (RA) patients by PHA stimulation. PBMC (2 × 10^5^/well) from RA patients (*n* = 25) and healthy controls (*n* = 20) were stimulated with PHA (5 μg/ml) in 200 μl culture media in 96-well plates. Supernatants were collected after 24 hrs and tested for IL-17 (**A**), IL-6 (**B**), IL-1β (**C**), TNF-α (**D**), IFN-γ (**E**), or IL-10 (**F**). The data are expressed as box plots. Each box represents the IQR. Lines inside the boxed represent the median. Whiskers represent the highest and lowest values. *P < 0.05, *versus* control (Mann–Whitney *U*-test).

A cytokine milieu that carries the inflammatory signature of RA is crucial in the differentiation and maintenance of pathogenic T cells, and in the dysfunction of Tregs 30. To determine cytokine profiles from the donor samples, we stimulated donor PBMC with PHA, and detected the cytokines in the supernatants by FlowCytomix. As shown in Figure 2, the PBMC of RA patients secreted higher levels of IL-17, IL-6, IL-1β and TNF-α than those of healthy controls (all *P* < 0.05), whereas IFN-γ levels decreased and IL-10 levels were not significantly changed.

**Figure 3:**
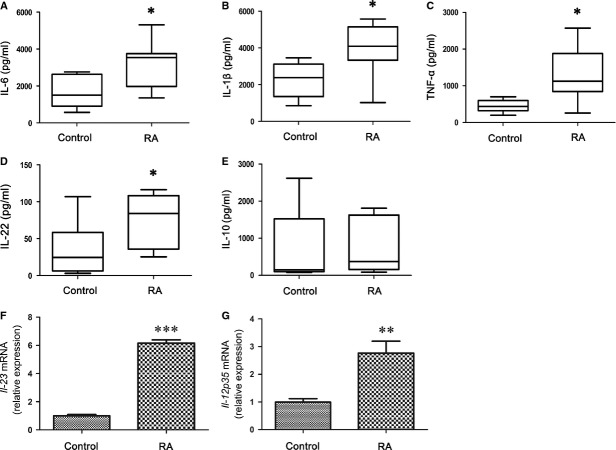
Increase in the production of pro-inflammatory cytokines from lipopolysaccharide (LPS)-stimulated peripheral blood mononuclear cells (PBMC) with rheumatoid arthritis (RA) patients. PBMC (2 × 10^5^/well) from RA patients (*n* = 25) and healthy control (*n* = 20) were stimulated with LPS (1 μg/ml) in 200 μl culture media in 96-well plates. Supernatants were collected after 24 hrs and tested for IL-6 (**A**), IL-1β (**B**), TNF-α (**C**), IL-22 (**D**), or IL-10 (**E**). The data are expressed as box plots. Each box represents the IQR. Lines inside the boxed represent the median. Whiskers represent the highest and lowest values. *P < 0.05, *versus* control (Mann–Whitney *U*-test). Cell pellets were collected after 24 hrs and mRNA expression of *Il-23* (**F**) or *Il-12p35* were detected (**G**). The expression of *Il-23* or *Il-12p35* mRNA in RA patients (*n* = 6) is shown as relative levels compared with healthy controls (*n* = 6). Data are expressed as the mean ± SEM. **P < 0.01 and ***P < 0.001, *versus* control (Student’s *t*-test).

Monocytes/macrophages are central to the pathophysiology of inflammation 31. Moreover, monocytes/macrophages from inflamed joints have been shown to induce the development of Th17, thus contributing to the amplification of inflammation 32. Because monocytes/macrophages were stimulated preferentially when PBMCs were stimulated with LPS *in vitro* 33, we stimulated PBMC with LPS then detected the cytokine secretion in the cell culture supernatants or the mRNA expression in the cell pellets. As shown in Figure 3, RA patients demonstrated higher levels of IL-6, IL-1β, TNF-α, IL-22 production, *Il-23* and *Il-12p35* mRNA expression than the healthy controls (all *P* < 0.05), whereas IL-10 levels were not significantly changed. These results suggest that both the lymphocytes and monocytes/macrophages from RA patients cause the up-regulation of pro-inflammatory cytokines, which results in the imbalance of Th17/Treg cells.

### Decreased expression of miR-21 in PBMC and CD4^+^ T cells of RA patients

**Figure 4:**
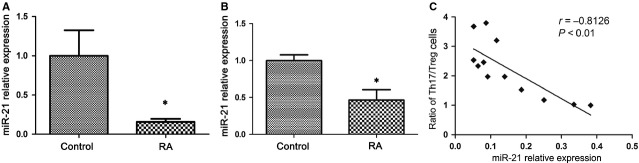
MiR-21 is decreased in peripheral blood mononuclear cells (PBMCs) and CD4^+^ T cells from rheumatoid arthritis (RA) patients. (**A**) PBMCs (3 × 10^6^) were isolated from RA patients (*n* = 25) and healthy control (*n* = 20), and miR-21 was quantified by real-time PCR. (**B**) Expression of miR-21 in sorted CD4^+^ T cells from RA patients (*n* = 5) and healthy control (*n* = 5) by real-time PCR. The expression of miR-21 in patients with RA is shown as relative levels compared with healthy controls. Data are expressed as the mean ± SEM. *P < 0.05, *versus* control (Student’s *t*-test). (**C**) The correlation between miR-21 expression level and the ratio of Th17/Treg cells in PBMC of RA patients (*n* = 12). The correlation was calculated by Spearman correlation analysis (Spearman *r* = −0.8126, *P* < 0.01).

Recently, miRNAs have emerged as key regulators of the immune system, being involved in lymphocyte selection and proliferation, and T-cell differentiation and homoeostasis. MiR-21 is specifically up-regulated in Tregs *versus* conventional T cells 19, suggesting that miR-21 may have an important role in Treg biology. MiR-21 is up-regulated in human Tregs, and can positively regulate Foxp3 expression 34. Given the defects of Treg cells in RA patients, we decided to compare the expression of miR-21 between PBMC and CD4^+^ T cells from RA patients and healthy controls. Indeed, as shown in Figure 4A and B, RA patients exhibited a significantly lower level of miR-21 than the healthy controls (*P* < 0.05). Furthermore, levels of miR-21 were negatively correlated with the ratio of Th17/Treg cells in PBMC from RA patients (Fig. 4C). These results suggest that the decrease in miR-21 expression may affect the development of Treg cells, and be involved in the imbalance of Th17/Treg cells in RA patients.

### Increased expression and activation of STAT3, and decreased expression of STAT5, p-STAT5 and *Foxp3* mRNA in RA patients

**Figure 5:**
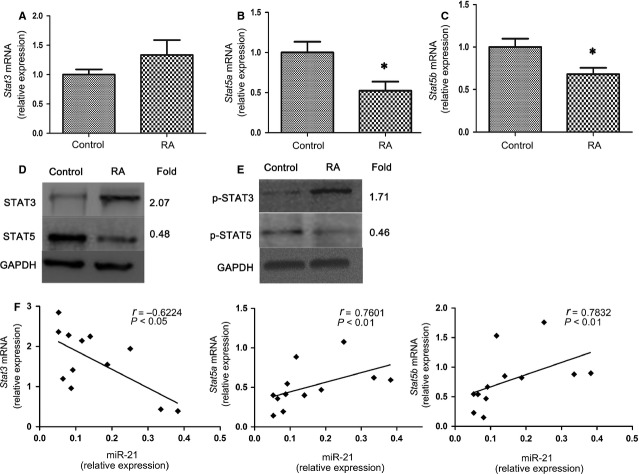
Increased expression and activation of STAT3, and decreased expression and activation of STAT5 in rheumatoid arthritis (RA) patients. (**A**) Total RNA from peripheral blood mononuclear cells (PBMC; 3 × 10^6^) in RA patients (*n* = 25) and healthy control (*n* = 20) were extracted. Real-time PCR analysis of the expression of *Stat3* (**A**), *Stat5a* (**B**), *Stat5b* (**C**) in patients with RA and healthy controls. The expression of these genes in patients with RA is shown as relative levels compared with healthy controls. Data are expressed as the mean ± SEM. *P < 0.05, *versus* control (Student’s *t*-test). (**D**) Total protein from PBMC (3 × 10^6^) in RA patients (*n* = 3) and healthy control (*n* = 3) was extracted. STAT3 and STAT5 protein expression was measured by Western blotting. (**E**) PBMC (3 × 10^6^) from RA patients (*n* = 3) and healthy control (*n* = 3) were stimulated with lipopolysaccharide (LPS) (200 ng/ml) for 24 hrs then extracted for total protein. p-STAT3 (Tyr705) and p-STAT5 (Tyr694) were measured by Western blotting. GAPDH was used as a loading control. The intensity of each band was analyzed using *LANE-1D* software (Beijing Sage Creation Science, China), and the ratio of the RA group to the control group for each item is presented as fold difference. Data are representative of three independent experiments. (**F**) Correlation of miR-21 expression with that of *Stat3*, *Stat5a* and *Stat5b* mRNA in PBMC of RA patients (*n* = 12). Statistical analysis was performed with Spearman correlation and *P* < 0.05 was considered to be significant.

**Figure 6:**
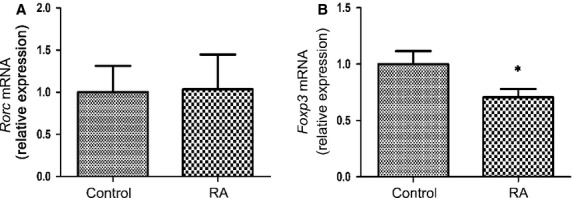
*Foxp3* mRNA is decreased in peripheral blood mononuclear cells (PBMC) from rheumatoid arthritis (RA) patients. PBMC (3 × 10^6^) was isolated from RA patients (*n* = 25) and healthy control (*n* = 20). *Rorc* (**A**) and *Foxp3* (**B**) gene expression was measured by qRT-PCR. The expression of *Rorc* and *Foxp3* in patients with RA is shown as relative levels compared with healthy controls. Data are expressed as the mean ± SEM. *P < 0.05, *versus* control (Student’s *t*-test).

A miR-21 target gene that was described previously is the gene encoding the primary transcript of STAT3 22,23, which plays a pivotal role in determining Th17 differentiation during RA 35,36. Furthermore, STAT5, as a key transcription factor in Treg cell differentiation, can regulate miR-21 expression, which is involved in the regulation of the balance between tolerance and immune activation 25. We thus assessed whether decreased miR-21 expression affected the expression and activation of STAT3 and STAT5 in RA patients. As shown in Figure 5, decreased mRNA expression of *Stat5a* and *Stat5b* mRNA was observed in RA patients compared with healthy controls (*P* < 0.05), whereas *Stat3* mRNA expression was not significantly changed (Fig. 5A–C). However, the expression of STAT3 protein was increased, whereas STAT5 protein was decreased in RA patients (Fig. 5D). Furthermore, the level of p-STAT3 was increased, whereas the expression of p-STAT5 was decreased in RA patients after LPS stimulation (Fig. 5E). Moreover, levels of miR-21 were negatively correlated with *Stat3* mRNA levels, while positively correlated with *Stat5a* and *Stat5b* mRNA levels in RA patients (Fig. 5F). Consistently, the mRNA expression of the Treg-specific transcription factor *Foxp3* was decreased in RA patients (*P* < 0.05); however, *Rorc* mRNA expression was not significantly changed (Fig. 6). The elevated expression and activation of STAT3, and reduced expression and activation of STAT5 in RA patients correlates with the increase in proportion of Th17 cells and decrease the frequencies of Treg cells in Figure 1. Thus, in RA, these results show a correlation between the decrease in miR-21, increase in Th17 and decrease in Treg frequencies by modulating STAT3/STAT5 expression and activation.

### LPS stimulation up-regulates miR-21 expression in healthy controls, but down-regulates miR-21 expression in PBMC from RA patients

**Figure 7:**
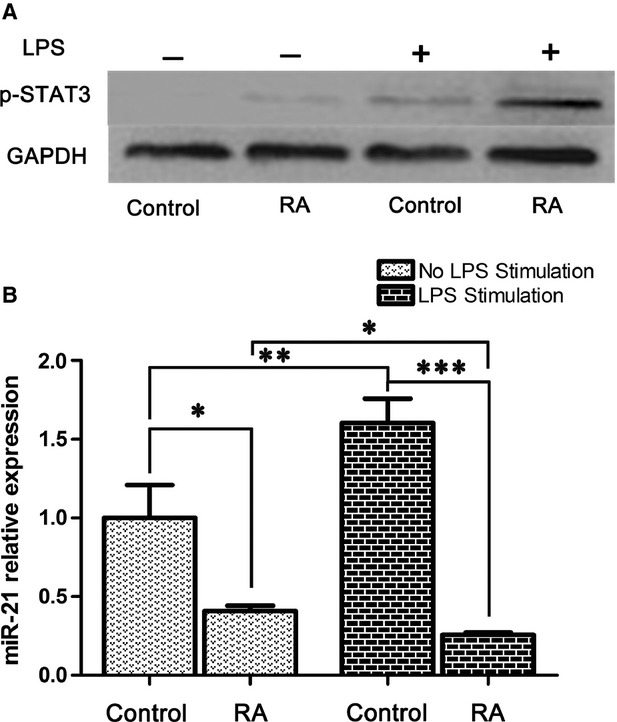
Lipopolysaccharide (LPS) stimulation up-regulated miR-21 expression in peripheral blood mononuclear cells (PBMC) from healthy controls, and down-regulated miR-21 expression from rheumatoid arthritis (RA) patients. PBMC (3 × 10^6^) from RA patients (*n* = 6) and healthy control (*n* = 6) with or without LPS (200 ng/ml) stimulation for 24 hrs were isolated for total protein or RNA. (**A**) p-STAT3 (Tyr705) activation level was measured by Western blotting. The results show a representative blot from three experiments. (**B**) MiR-21 was quantified by real-time PCR. The expression of miR-21 is shown as relative levels compared with the group without LPS stimulation from healthy controls. Data are expressed as the mean ± SEM. *P < 0.05; **P < 0.01; ***P < 0.001 (Student’s *t*-test).

It has been reported that STAT3 also regulates miR-21 expression 24. LPS stimulation induced the up-regulation of p-STAT3 in PBMC from RA patients and healthy controls, with the increase in p-STAT3 in RA patients more significant than the healthy controls (Fig. 7A). We next tested the correlation between the induction of p-STAT3 and the expression of miR-21. As shown in Figure 7B, LPS treatment resulted in a higher level of miR-21 in PBMC from healthy controls (*P* < 0.01). However, LPS stimulation induced a statistically significant decrease in miR-21 expression in RA patients (*P* < 0.05). Decreased miR-21 expression was observed in PBMCs with or without LPS stimulation from RA patients compared with healthy controls (*P* < 0.05 or *P* < 0.001). These results suggest that LPS stimulation induces p-STAT3 and miR-21 expression in healthy control cells, whereas in PBMC of RA patients down-regulation of miR-21 expression following LPS induction allows for significantly greater STAT3 activation.

## Discussion

The imbalance of Th17/Treg cells has been reported to contribute to several inflammatory autoimmune diseases, including RA. However, the mechanisms governing the imbalance of Th17/Treg cells resulting in autoimmune arthritis remain unclear. Consistent with previous studies 37,38, our study also demonstrated the increased ratio of CD3^+^CD4^+^IL-17^+^ Th17 cells to CD4^+^CD25^+^Foxp3^+^ Treg cells in the PBMC of RA patients. Over the course of RA, a cytokine milieu is built progressively in inflamed synovia, which carries the inflammatory signature of RA for promoting the differentiation of pathogenic T cells 30. In humans, Th17 cells can be induced by IL-6, IL-1β and TNF-α 39, all of which are abundant in the inflamed joint in patients with active RA, and are directly involved in the destruction of cartilage and bone 3,12. Consistent with these studies, our study demonstrated that PBMCs from RA patients stimulated by PHA could secrete higher levels of IL-17, IL-6, TNF-α and IL-1β compared with healthy donors. Although it is generally considered that Th1 and Th17 cells play an important role in the progression of RA 40,41, we found that the level of IFN-γ mainly produced by Th1 cells from RA patients was significantly decreased compared with the healthy controls. These results are consistent with another study demonstrating that patients with RA showed a lower secretion of IFN-γ than healthy controls; however, the secretion of IFN-γ was increased after infliximab treatment 42. Furthermore, Evans and colleagues reported that *in vivo* activated CD14^+^ monocytes derived from the inflamed joints of RA patients spontaneously and specifically induce a Th17, but not a Th1 or Th2 response 32. Thus, this finding supports the notion that Th17, rather than Th1 cells, is the pathogenic culprit in RA 41.

The quantification of cytokines from LPS-stimulated PBMCs in RA patients also demonstrated a significantly increase in pro-inflammatory cytokines, including IL-6, TNF-α, IL-1β, and IL-22, *Il-23* and *Il-12p35* mRNA levels, compared with the healthy controls. These results indicate that Th17 responses might be induced by LPS-stimulated monocytes/macrophages and the differentiation Th17 was orchestrated by producing the above inflammatory cytokines in RA patients. Moreover, it has been suggested that exposure to inflammatory cytokines, including IL-6 43, TNF-α 44 and IL-1β 45, can inhibit the function of Treg cells by limiting Foxp3 expression. The down-regulation of Foxp3 expression not only affects the function of Treg cells but also impairs the generation of Treg cells, as Foxp3 is indispensable in the development and function of Treg cells 46. Thus, our study suggests that high levels of inflammatory cytokines in PBMCs carry the inflammatory signature of RA and are crucial in the differentiation and maintenance of pathogenic Th17 cells as well as the dysfunction and inhibition of Treg cells.

It has been reported that miR-21 has an important role in Treg biology 34, and is involved in the development of a variety of inflammatory diseases 14–16. We wanted to know the expression of miR-21 in RA patients with defective Treg cells. As expected, we did see a significantly decrease in miR-21 expression in the PBMCs and CD4^+^ T cells of RA patients compared with the healthy control. MiR-21 can positively regulate Foxp3 expression 34; our study indicated that the decrease in miR-21 may be a determinant in the reduction in Treg cells in RA, and the lack of control in terms of Th17 cell development. Consistently, levels of miR-21 were negatively correlated with the ratio of Th17/Treg cells in PBMCs from RA patients. Our results are consistent with previous work which showed reduced levels of miR-21 in synovial tissues 28 or CD4^+^ T cells from synovial fluid of RA 47 *via* microarray. However, Wang and colleagues reported that miR-21 was up-regulated in the whole blood from RA patients 48. Whether these differences are because of different samples, detection methods, or disease progression and stage, further studies remain to be investigated. Furthermore, the expression of miR-21 in other rheumatic disorders was also found up-regulated in T cells both in patients with lupus and lupus-prone MRL/lpr 49 and B6.Sle 123 mice 16. The differences in miR-21 expression in rheumatic disorders might be related to the differences in host immune system, differences in disease models and differences in pathogenesis. A greater understanding of the exact roles of miR-21 in rheumatic diseases will be valuable in the development of miR-21-targeted therapy strategy.

There is evidence that miR-21 is involved in the regulation of STAT3 22,23 and STAT5 25, which are regulators of Th17 and Treg cell differentiation, respectively 21. Unexpectedly, we found that *Stat3* mRNA slightly but not significantly increased, whereas the mRNA levels of *Stat5a* and *Stat5b* were significantly decreased in RA patients compared with the healthy control. The decrease in mRNA levels of *Stat5a* and *Stat5b* may explain the deficiency of Treg cells in RA patients. However, STAT3 and p-STAT3 protein levels were increased, whereas STAT5 and p-STAT5 was decreased in RA patients, which was consistent with the enrichment of Th17 cells and deficiency of Treg cells. Although we did not detect the direct link between miR-21 and STAT3 or STAT5, Kim and colleagues reported that overexpression of miR-21 in human adipose tissue-derived mesenchymal stem cells decreased both protein and mRNA levels of STAT3, whereas inhibiting miR-21 with 2′-O-methyl-antisense RNA increased these levels 22. We therefore hypothesize that the decrease in miR-21 may increase the expression and activation of STAT3 in RA patients, thus promoting Th17 cell differentiation. Iliopoulos and colleagues revealed that PD-1 inhibition resulted in enrichment of STAT5 binding in the miR-21 promoter region, and promoting miR-21 expression 25. Further analysis is needed to determine exactly how the regulation of the expression and activation of STAT3, STAT5 and miR-21 in RA patients is globally controlled, and how each factor interplays to exhibit the disease profile in these immune cells.

Consistent with the current work, Ju and colleagues reported that the expression of STAT3 increased in proportion to the severity of synovitis in RA synovial tissue using immunostaining methods, and transfection with STAT3 siRNA in CD4^+^ T cells prevented Th17 differentiation from RA peripheral blood and synovial fluid but increased the proportion of Treg cells. In contrast, inhibition of STAT5, increased the proportion of Th17 and reduced Treg cells 35. Consistently, we found that the mRNA of *Foxp3*, the transcription factor controlling regulatory T cell development 50, was decreased in PBMC from RA patients. However, we did not find that the level of *Rorc* mRNA, the master regulator that directs the differentiation program of Th17 cells 51, was increased in RA patients. These results are consistent with a previous study demonstrating that IL-21 promotes *Rorc* mRNA expression in both healthy control and RA PBMC, although they did not compare the expression of Rorc mRNA between the healthy control and RA patients 52; it also supports the notion that there is a limited role for the master regulator RORγt in initiating the establishment of active enhancer networks in Th17 lineages 53.

Apart from the regulation of Stat3 expression by miR-21, multiple studies have shown that IL-6 or IL-21, acting on Stat3, can positively regulate miR-21 transcription. For example, stimulation of Sézary cells or healthy CD4^+^ T cells with IL-21 results in the strong activation of STAT3, and subsequent up-regulation of miR-21 expression 54. MiR-21 induction by IL-6 is strictly Stat3 dependent 24. As LPS stimulation can induce the increase in STAT3 activation from both healthy control and RA patients, we wanted to analyse miR-21 expression after LPS stimulation. Expectedly, miR-21 expression was significantly increased after LPS stimulation from healthy controls. Although the increase in p-STAT3 in RA patients was more apparent than in the healthy controls, LPS treatment decreased the expression of miR-21 from RA patients. We therefore hypothesized that miR-21 may act as part of negative feedback loop in the normal setting, such that miR-21 would be induced in response to ongoing inflammation and would in turn regulate Th17 inflammation by directly targeting STAT3. However, miR-21 levels decrease significantly in RA patients, suggesting that this feedback loop is dysregulated in RA patients and may contribute to the imbalance of Th17 and Treg cells, and induction of chronic inflammation in RA.

In addition, it has been reported that miR-21 negatively regulates macrophage activation by targeting IL-12p35 and PDCD4 55. Thus, the expression of *Il-12p35* and *Pdcd4* mRNA in RA patients and healthy controls were detected. Indeed, increased mRNA expression of *Il-12p35* mRNA was observed in RA patients compared with healthy controls, but *Pdcd4* mRNA expression was not significantly changed (Fig. S1). The elevated expression of *Il-12p35* in RA patients might be related to the decrease in miR-21 in RA patients. The differences in *Pdcd4* expression in RA patients might be related to the differences in host immune system, differences in disease models, and differences in pathogenesis.

**Figure 8:**
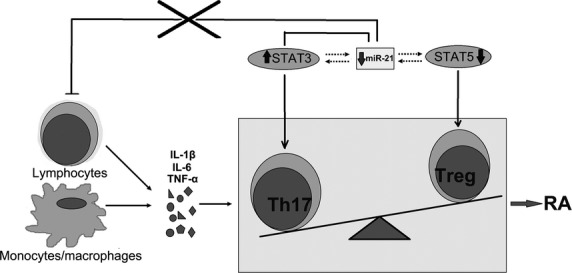
Schematic diagram showing the hypothetical role of miR-21 in rheumatoid arthritis (RA) patients. In the normal setting, miR-21 can inhibit inflammation by positively regulating Treg cell development or negatively regulating STAT3. However, decreased levels of miR-21 may increase the expression and activation of STAT3, and simultaneously reduce the expression and activation of STAT5, promoting the Th17 cell differentiation while suppressing Treg cell development during chronic inflammation in RA.

In conclusion, this article demonstrates that the imbalance of Th17/Treg cells exists in RA patients. This imbalance may be induced by the high levels of inflammatory cytokines from lymphocytes or LPS-stimulated monocytes/macrophages to orchestrate Th17 cell differentiation, and simultaneously inhibit Treg cell development in RA patients. The decrease in miR-21 levels may be involved in the induction of the imbalance of Th17/Treg cells during Th cell development in RA patients. We speculate that miR-21 can inhibit inflammation by positively regulating Treg cell development 35,56 or negatively regulating STAT3 22,23 under normal settings. However, decreased miR-21 may increase the expression and activation of STAT3, and simultaneously inhibit the expression and activation of STAT5, promoting Th17 differentiation while suppressing Treg development during chronic inflammation in RA (Fig. 8). MiR-21 may serve as a novel regulator in T cell differentiation and homoeostasis; however, the functional role of miR-21 in regulating the development of Th17 and Treg cells warrants further investigation.

## Supporting Information

Figure S1 *Il-12p35* mRNA is increased in PBMC from RA patients. PBMC (3 × 10^6^) was isolated from RA patients (*n* = 6) and healthy control (*n* = 6). *Il-12p35* (A) and *Pdcd4* (B) gene expression was measured by qRT-PCR. The expression of *Il-12p35* and *Pdcd4* in patients with RA is shown as relative levels compared with healthy controls. Data are expressed as the mean ± SEM. ***P* < 0.01, *versus* control (Student’s *t*-test).
