# Supplementary figures and images for: Decreased expression of microRNA-21 correlates with the imbalance of Th17 and Treg cells in patients with rheumatoid arthritis
Source: J Cell Mol Med. 2014 Aug 28;18(11):2213–24. doi: 10.1111/jcmm.12353 (PMC4224555; doi:10.1111/jcmm.12353)

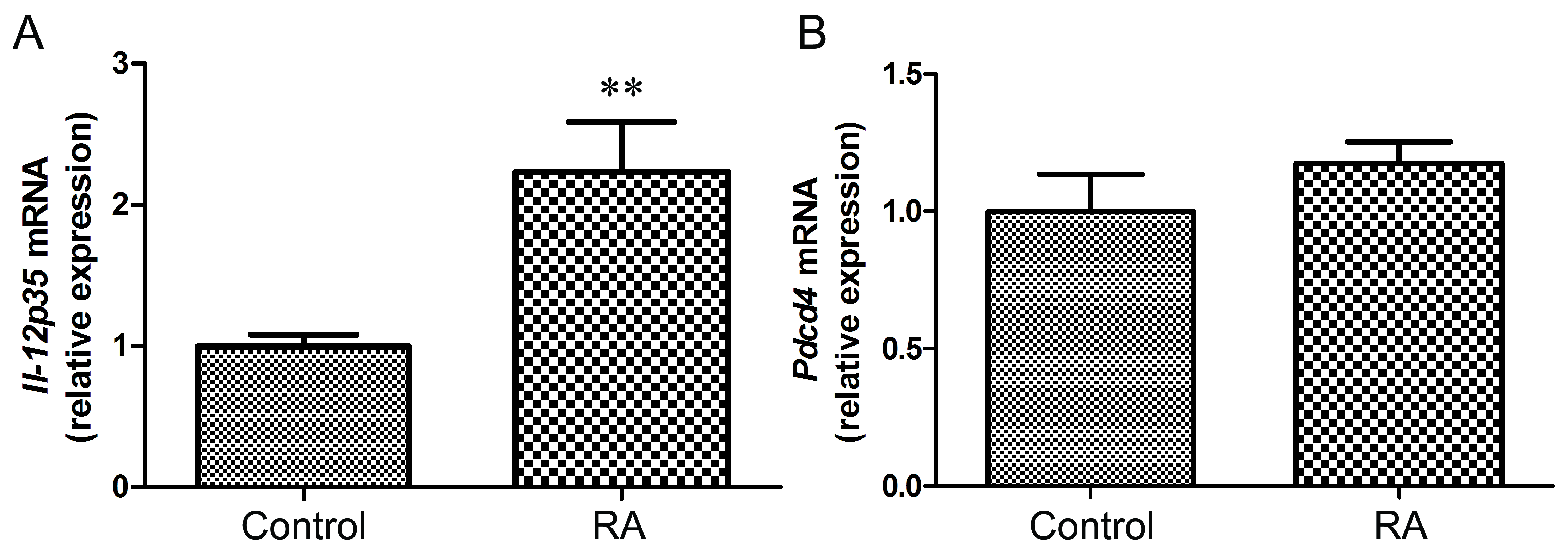

Supplement: Figure S1 — Il-12p35 mRNA is increased in PBMC from RA patients. PBMC (3 × 10^6^) was isolated from RA patients (n = 6) and healthy control (n = 6). Il-12p35 (A) and Pdcd4 (B) gene expression was measured by qRT-PCR. The expression of Il-12p35 and Pdcd4 in patients with RA is shown as relative levels compared with healthy controls. Data are expressed as the mean ± SEM. **P < 0.01, versus control (Student’s t-test). [file jcmm0018-2213-sd1.tif]
